# Portal hypertensive biliopathy: Can we prevent it?

**DOI:** 10.4103/0971-9261.69132

**Published:** 2010

**Authors:** K. L. N. Rao, B. R. Thapa

**Affiliations:** Department of Pediatric Surgery, Postgraduate Institute of Medical Education and Research, Chandigarh, India; 1 Department of Pediatric Gastroenterology, Postgraduate Institute of Medical Education and Research, Chandigarh, India

In developing countries, extrahepatic portal vein obstruction (EHPVO) accounts for the majority of cases of portal hypertension.[1] In general, endoscopic treatment of varices is capable of taking care of acute variceal bleeding as well as obliterating the varices in the long run.[2] However, frequent follow-ups are required permanently thereafter, to watch for the development of various other sequelae. Various shunt surgeries are accepted as indications for massive splenomegaly/hypersplenism, ectopic varices, gastropathy, enteropathy, logistic/economic reasons or as a one-time therapy for the disease.[3,4] Quality of life and somatic growth deficiency are also gradually being recognized as situations demanding surgery.[5,6]

Initially, it was believed that children with extrahepatic portal hypertension (EHPH) would grow out of their bleeds. However, it is now clear that this is not true, and the natural history of EHPH in relationship to the frequency of complications requiring further and more aggressive treatment modalities such as shunt surgeries is not known.

Portal biliopathy[7,8] refers to abnormalities of the extrahepatic, intrahepatic bile ducts and the gall bladder wall in patients with portal hypertension. Pain/jaundice due to strictures, dilatations, irregular walls, stone formation, and para-choledochal collaterals are the result. It is believed that the external pressure of the portal cavernoma or collaterals and/or ischemia is the causative factor. Biliopathy is quite a difficult complication to treat and may end up in secondary biliary cirrhosis and adversely affect the life of the patient in the long run. The fact to note is that it usually takes two to three decades to develop portal biliopathy, usually in adulthood. ERCP[9] or MR angiography can pick up the tell-tale evidence of biliopathy even before the onset of jaundice, pain or even raised liver enzymes in the blood.

For surgeons, who have operated on children and adults with EHPH, there is no doubt that surgery is far easier on children than adults because of worsening collaterals, inflammatory fat deposition, ischemic changes in the biliary tree, and difficult access to the portal venous tree for surgical maneuvers, with the passage of time. Is there a need for a positive effort by the clinicians to pick up initial biliopathy changes in the early second decade of life and prevent the development of full-blown biliopathy by adopting early shunt surgery? Portosystemic shunting procedures are definitive procedures to relieve the biliary stasis and prevent further stone formation.[8] All the clinicians who are caring for patients with portal hypertension need to deliberate and throw light on this issue.
